# Risk Factors for Infection with Different Hepatitis C Virus Genotypes in Southern Brazil

**DOI:** 10.1100/2012/946954

**Published:** 2012-05-22

**Authors:** Marisa Lúcia Romani Paraboni, Marina Dallagasperina Sbeghen, Fernando Herz Wolff, Leila Beltrami Moreira

**Affiliations:** ^1^Universidade Regional Integrada Campus Erechim, Erechim, Brazil; ^2^National Institute of Science and Technology for Health Technology Assessment (IATS)-CNPq, Porto Alegre, Rio Grande do Sul, Brazil; ^3^Post-Graduate Program in Medicine (Epidemiology)-Federal University of Rio Grande do Sul, Porto Alegre, Brazil; ^4^Pharmacology Department, Ciências Básicas da Saúde Institute, Federal University of Rio Grande do Sul, Brazil

## Abstract

*Objectives*. To investigate the proportion of different genotypes in countryside microregions in southern Brazil, and their association with risk factors. *Methods*. Cross-sectional study including a convenience sample of patients who tested positive for HCV-RNA and were referred to a regional health center for genotyping, from December 2003 to January 2008. Data were obtained through the National Disease Surveillance Data System, from laboratory registers and from patient charts. Identification of genotypes was carried out using the Restriction Fragment Length Polymorphism “in house” technique. Independent associations with genotypes were evaluated in multinomial logistic regression and prevalence rates of genotypes were estimated with modified Poisson regression. *Results*. The sample consisted of 441 individuals, 41.1 ± 12.0 years old, 56.5% men. Genotype 1 was observed in 41.5% (95% CI 37.9–48.1) of patients, genotype 2 in 19.3% (95% CI 15.0–23.6), and genotype 3 in 39.2% (95% CI 35.6–43.0). HCV genotype was significantly associated with gender and age. Dental procedures were associated with higher proportion of genotype 2 independently of age, education, and patient treatment center. *Conclusions*. The hepatitis C virus genotype 1 was the most frequent. Genotype 2 was associated with female gender, age, and dental procedure exposition.

## 1. Background

Hepatitis C virus (HCV) was identified in 1989 and has been considered a major cause of chronic liver disease worldwide [1]. There is a great variability in its geographical distribution, associated to the degree of nation development. High prevalence is found in Africa and Asia, in opposite to low-prevalence areas localized in industrialized nations in North America, north and west Europe, and Australia [2–4]. In Brazil, according to the World Health Organization, the estimated prevalence ranges from 2.5 to 4.9% [5].

Transmission of HCV has been mainly related to intravenous drug use since blood products transmission has decreased in most developed countries. On the other hand, contaminated injection equipment appears to be the major risk factor for HCV infection in several countries and sharing personal hygiene objects might explain the transmission of virus C to those not infected by the usual routes [6]. The distribution of different genotypes also varies according to the studied population and viral transmission risk factors. In studies from Spain there is a predominance of genotypes 1a and 1b [7, 8] while in other European regions genotype 2 is usually the most prevalent [4, 9–11]. Genotype 1 predominates in Central America [12], and in Latin-American countries such as Argentina [13, 14] and Venezuela [15] genotypes 1 and 2 account for 90% of cases. In Brazil, genotypes 1 and 3 are the most frequent [16, 17], but in Da Silva et al. study [18] almost half of the hepatitis C patients from South of Brazil were infected by genotypes 2 and 3. In this study we investigated the proportion of different genotypes in countryside microregions of a state in southern Brazil, and their association with sociodemographic characteristics and HCV infection risk factors.

## 2. Methods

A cross-sectional study included a nonprobabilistic sample of patients under followup at the HCV program of Brazilian Public Health System, in countryside cities of southern Brazil. Patients from the Brazilian Public Health System, who tested positive for anti-HCV, were referred for genotyping, from December 2003 to January 2008, to a main regional health center in the southernmost state of Brazil. Genotyping was routinely performed to choose the recommended treatment according HCV genotype. HCV-RNA was carried out as a confirmatory test and the samples of all patients genotyped at the central laboratory in the period were included consecutively.

Retrospective data collection was carried out and included demographic and socioeconomic characteristics, exposure and behavioral risks factors. Data were obtained through the National Disease Surveillance Data System (SINAN), laboratory registers and from patient charts at their cities of origin. Researchers were trained before data collection start, and quality control was performed by random repetition of data collection in 10% of the sample.

Identification of genotypes was carried out using the Restriction Fragment Length Polymorphism “in house” technique [19], which uses universal primers and real time-polymerase chain reaction to amplify specific genomic sequences and it compares to strip hybridization. Next, DNA fragments of different sizes are generated by enzymatic restriction digestion, which recognizes specific cleavage sites for each genotype. This method allows differentiation of genotypes 1, 2, 3, 4, 5, and 6 [20]. Preanalytic quality control included the sample and the reagents preparation, amplification and detection, and environment control.

The study has been performed according to the World Medical Association Declaration of Helsinki and approved by the Ethics Committee of Passo Fundo University, Brazil. Regional health centers consented with the study, and an agreement on data use was signed.

A sample size of 384 individuals was necessary to estimate a genotype 1 prevalence of 50%, with a 95% confidence interval and a 10% prevalence variation. Considering the less expected genotype 2, 292 patients would be necessary to determine 5% ±5 prevalence.

Data was described using frequency and central tendency measures, and 95% confidence intervals were calculated when applicable. Associations between risk factors and genotype were analyzed using chi-square. To identify independent associations with genotype we used multinomial logistic regression. Models were tested taking into account variables with a *P* value < 0.20 on the crude analysis, and those that remained independently associated with the genotype were included in the final model. To explore risk factors that could be associated with the prevalence of genotype 2, genotypes 1 and 3 were grouped in the reference category, and adjusted prevalence ratio was calculated using modified Poisson regression, in a model that included the same variables as the multinomial logistical regression.

## 3. Results

From December 1st 2003 to January 28th 2008, 411 patients HCV-RNA positive were submitted to genotyping. Mean age was 41.1±12.0 years, 56.5% were men and most patients (73.9%) were from the health regional coordination of Passo Fundo, a medium size city in south of Brazil.

**Figure 1:**
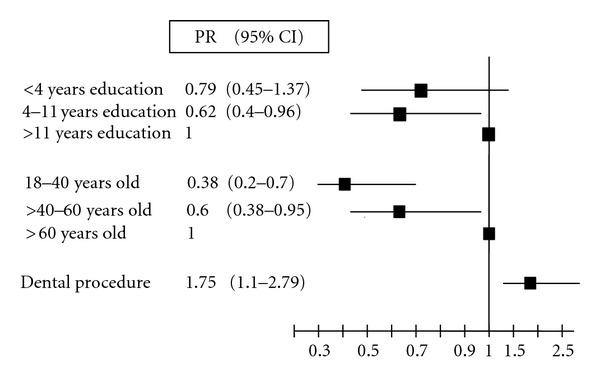
Prevalence ratio for genotype type 2 in patients with hepatitis C in southern Brazil.

The proportions of genotypes were 41.5% (95% CI 37.9–48.1, *n* = 183) for type 1 (55 subtype 1a and 41 subtype 1b), 19.3% (95% CI 15.0–23.6, *n* = 85) for type 2, and 39.3% (95% CI 35.6–43.0, *n* = 173) for type 3. There was a difference in genotype 2 distribution when comparing health centers, with higher genotype 2 prevalence in the largest city (22.7% versus 9.6%; *P* = 0.007).  Table 1 presents the genotype distribution according to sociodemographic characteristics and risk factors. Data regarding genotype, gender, and age were obtained for all patients. Some other variables were lost due to incomplete notification form filling. Genotype 1 was the most prevalent among men, while among women it was genotype 3 (*P* = 0.028). Patients carrying genotype 2 were older (52.2 ± 12.8 years for type 2, versus 43.6 ± 11.2 years for type 1 and 45.6 ± 11.5 years for type 3; *P* < 0,001), and the prevalence of nonwhite subjects was lower than in other groups (3.6% in type 2, versus 13.1% in type 1 and 6.6% in type 3). Among several established risk factors for HCV infection, use of intravenous and inhalation drugs, surgical treatment, and dental procedures were associated to the genotype (Table 2). On the multinomial regression, older age, history of dental procedure, and higher education increased the risk for infection by genotype 2 (Table 3). Adjusted prevalence ratios are shown in Figure 1. Dental procedure was associated with higher prevalence of genotype 2 independently of age, education and of which treatment center patient came from.

## 4. Discussion

In this study we observed prevalence of genotypes 1, 2, and 3 of 41.5%, 19.3%, and 39.2%, respectively, which are not the same as observed in other regions of the country and the world. A study conducted in Spanish health centers [7] described a higher proportion of genotype 1 (65.4%) and only 3.1% of genotype 2. In Poland [9] there is a genotype 1 percentage much similar to the one found in this study, but with a higher genotype 2 (37.8%) and a lower genotype 3 (23.4%) proportion. In south India genotype 1 was found in only 18.8% of population, and genotype 3 was higher (62.2%) than in this study [24]. Of 284 samples from the III *National Health and Nutrition Examination Survey*, conducted in North and Central America, 275 were genotyped [25]. As in our study, genotype 1 was the most common one, but with an even higher prevalence (78.2%; 1a = 51.6% and 1b = 26.6%). Genotypes 2 (12.7%; 2a = 2.9 and 2b = 9.8) and 3 (6.2%) were less common, and genotypes 4 and 6 were also detected (1.1% and 1.8%, resp.). In Latin America countries such as Argentina and Venezuela, genotypes 1 and 2 account for almost 90% of cases [13–15].

In Brazil (Table 4), a study [21] with 1.668 samples collected between 1995 and 2000 in laboratories from different regions detected genotypes 1 (64.9%), 2 (4.6%), 3 (30.2%), 4 (0.2%), and 5 (0.1%). Proportions of each genotype were significantly different according to each region, but genotype 1 was always the most common (51.2% to 74.1%). The south region showed a lower proportion of genotype 1, when compared to other regions (51.2% versus 57.0 to 74.1%, *P* = 0.001), and a higher proportion of genotype 3 (40.3% versus 24.7 to 31.6%). This pattern of distribution is similar to the one we found in the present study. Another study [22] with patients from several healthcare centers in Brazil included more participants, being 81% from public institutions and 19% from private practice. Patients mean age was 46 years, 62% were male and 80% were white. Genotypes 1 (64%), 2 (1.3%), 3 (33%), and 4 (1.7%) were identified. Most patients were from the south and southeast regions, and only 4% were from the northeast. Once again, there is a higher proportion of genotype 3 in the south compared to north and northeast (44% versus 27% and 26%). Genotype 1 was found in 51% of samples from southeast region and in 71% of samples from the northeast. Genotype 2 was detected in only 2% of samples from northeast and southeast, and in 5% of samples from the south. In the central-west region the prevalence of genotype 2 was higher (11.4%), but it was still lower than the one we found in our study (19.3%), which included only countryside cities. Our findings also differ from two studies conducted in Porto Alegre, the capital of state. The first was a retrospective study with 400 patients under treatment [23] from 1999 to 2000, which described a similar genotype distribution among men and women and a low prevalence of genotype 2 (41.3% of genotype 1, 5.0% of genotype 2, and 53.7% of genotype 3). In the second study [6], genotype 1 was diagnosed in 81.5% of coinfected outpatients in a HIV/AIDS reference center of the Brazilian public health system and was associated with male gender. The study recorded only 1.7% of genotype 2. The higher prevalence of genotype 3 among women found in our study can be explained by the older age of this group, since genotype 3 showed a positive and independent association with age.

 In Venezuela, increasing genotype 2 prevalence was described, rising from 26% in 1994–96 to 41% in 2005-06 [15]. A study conducted in Yucatãn, Mexico [26], described a genotype 2 prevalence of 33.3% significantly associated with family history of liver disease. Factors positively associated with genotype 2 in our study were age, higher education, and history of dental procedures. These characteristics may indicate a higher socioeconomic level, suggesting a specific risk profile. Genotype 2 among patients from Passo Fundo may be associated to socioeconomic and cultural differences in comparison to other smaller cities in the region. Although dental treatment is a known risk factor for hepatitis B [27], dental procedures include surgical treatment which represents a potential for HCV transmission through contaminated injection equipment. Even oral cavity examination might be involved, since it was found that sharing personal hygiene objects might explain the transmission of HCV [6].

Viral load has crucial relevance on the viral transmission. On crude analysis, there was a statistically significant association between genotype and risk factors for HCV transmission, as the use of injection and inhalation drugs, surgical treatment and dental procedures. Despite infected patients with history of previous exposition to those factors being more commonly infected by genotype 1, genotype 2 was relatively more frequent in participants with history of dental procedure (26%) in comparison to other risk factors (Table 1). These findings differ from studies with drug users in Poland [9] and Siberia [28], which recorded association between dental procedures and HCV genotype 1. But it agrees with a study from Lybia that recorded high incidence of the genotype 2 after surgery and dental procedure [29].

HCV genotype is a predictive factor to antiviral treatment response. There are clear evidence [30] indicating that genotypes 1 and 4 are associated to poor interferon response, either in single therapy or combined with ribavirin, the opposite being true for genotypes 2 and 3 treated for 24 weeks. Best treatment results, measured by viral parameters, are reached within 48 weeks for patients with genotype 2 or 3, while patients with genotype 1 need one year of treatment. Therefore, long-term benefits of HCV treatment may be estimated based on the characteristics of the treated population.

Since HCV treatment has high costs and is provided by the public health care system for all Brazilian citizens, we believe that almost all HCV positive patients from that regional population were included in the studied sample, but they may be representative of the lower income population. Although disease register is mandatory, occasionally the form was incompletely filled, causing loss of epidemiologic information and a limitation of our study. The method limitation to identify genotype subtypes deserves mention, but this was not an aim of the study and it is still the method performed in the Brazilian health system and the only one offered by the public system, in the place where the study was conducted.

In conclusion, in a region from south Brazil the most common HCV genotype was type 1, followed by type 3, in accordance to previous reports, but the proportion of genotype 2 was higher than expected and was significantly associated to history of dental procedures and older age.

## Figures and Tables

**Table 1:** Sociodemographic characteristics of hepatitis C virus infected patients according to genotype.

Characteristics	N*	Genotype 1	Genotype 2	Genotype 3	*P* value
		*N* (%)	*N* (%)	*N* (%)	
Male	249	116 (46.6)	40 (16.1)	93 (37.3)	0.028
Female	192	67 (34.9)	45 (23.4)	80 (41.7)	

White color	389	152 (39.1)	81 (20.8)	156 (40.1)	0.012
Nonwhite color	38	24 (63.1)	3 (7.9)	11 (28.9)	

Education					0.079
0–3 years	54	24 (44.4)	14 (25.9)	16 (29.6)	
4–11 years	220	89 (40.5)	38 (17.3)	93 (42.3)	
College	57	22 (38.6)	18 (31.6)	17 (29.8)	

Age	441				<0.001
18–40 years		79 (51.1)	18 (11.6)	58 (37.4)	
>40–60 years		92 (39.9)	51 (21.6)	93 (39.4)	
>60 years		12 (24.0)	16 (32.0)	22 (44.0)	

**Table 2:** Genotype distribution according to risk factors among hepatitis C virus-infected patients from the countryside of southern Brazil (*N* and %).

Risk factors	N*	Genotype 1	Genotype 2	Genotype 3	*P* value*
Blood transfusion	397				0.160
Yes		50 (36.5)	33 (24.1)	54 (39.4)	
No		118 (45.4)	46 (17.7)	96 (36.9)	

Use of IV medication	393				0.199
Yes		68 (41.7)	39 (23.9)	56 (34.4)	
No		95 (41.3)	40 (17.4)	95 (41.3)	

Acupuncture	393				0.726
Yes		4 (30.8)	3 (23.1)	6 (46.2)	
No		163 (41.5)	79 (20.1)	151 (38.4)	

Hemodialysis	390				0.510
Yes		4 (30.8)	2 (15.4)	7 (53.8)	
No		157 (41.6)	77 (20.4)	143 (37.9)	

Exposure to blood or organic secretions	391				0.119
Yes		26 (31.7)	19 (23.2)	37 (45.1)	
No		137 (44.3)	58 (18.8)	114 (36.9)	

Tatoo	393				0.207
Yes		25 (46.3)	6 (11.1)	23 (42.6)	
No		138 (40.7)	73 (21.5)	128 (37.8)	

Use of injection drugs	394				0.032
Yes		38 (53.5)	7 (9.9)	26 (36.6)	
No		129 (39.9)	71 (22.0)	123 (38.1)	

Surgical treatment	393				0.023
Yes		81 (39.5)	52 (25.4)	72 (35.1)	
No		82 (43.6)	27 (14.4)	79 (42.0)	

Piercing	393				0.643
Yes		4 (40.0)	1 (10.0)	5 (50.0%)	
No		159 (41.5)	78 (20.4)	146 (38.1)	

Inhalation drug	389				0.028
Yes		32 (53.3)	5 (8.3)	23 (38.3)	
No		130 (39.5)	73 (22.2)	126 (38.3)	

Dental procedure	394				0.010
Yes		72 (36.7)	51 (26.0)	73 (37.2)	
No		92 (46.5)	28 (14.1)	78 (39.4)	

Transplant	391				0.287
Yes		1 (25.0)	0 (0.0)	3 (75.0)	
No		161 (41.6)	79 (20.4)	147 (38.0)	

Percutaneous accident	389				0.979
Yes		7 (43.8)	3 (18.8)	6 (37.5)	
No		154 (41.3)	75 (20.1)	144 (38.6)	

*Chi-square test; IV: intravenous.

**Table 3:** Risk factors for genotype 1 and for genotype 3 compared to genotype 2 in patients with HCV infection in the countryside of southern Brazil.

	RR	95% CI	P*
Genotype 1			

Age	0.94	0.91–0.96	<0.001

Education			
0–3 years	2.38	0.81–6.57	0.095
4–11 years	1.94	0.89–4.25	0.096
≥12 years	1		

Dental procedure			
Yes	0.37	0.19–0.70	0.002
No	1		

Genotype 3			

Age	0.95	0.92–0.97	<0.001

Education			
0–3 years	1.98	0.69–5.73	0.206
4–11 years	2.69	1.21–5.98	0.016
≥12 years	1		

Dental procedure			
Yes	0.48	0.25–0.93	0.029
No	1		

*Multinomial regression adjusted for age, education, skin color, and dental procedure.

**Table 4:** Proportional HCV genotype distribution according to Brazilian studies.

Study	*N*	Region	Genotypes (%)				
			1	2	3	4	5
Campiotto et al. [21]	85	North	74.1	1.2	24.7	0	0
	237	Northeast	66.6	2.9	30.4	0	0
	79	Center-west	57.0	11.4	31.6	0	0
	1111	Southeast	59.6	3.8	28.4	0.3	0.2
	176	South	51.2	8.0	40.3	0	0
	1688	Total	64.9	4.6	30.2	0.2	0.1

Focaccia et al. [22]	121	Northeast	71.0	2.0*	27.0	NI	0
	705	Southeast	72.0	2.0*	26.0	NI	0
	522	South	51.0	5.0*	44.0	NI	0
	1348	Total	64.0	1.3	33.0	1.7	0

Alves et al. [23]	400	Porto Alegre-RS	41.3	5.0	53.7	0	0

Wolff et al. [6]	173	Porto Alegre-RS	81.5	1.7	16.2	0	0

*Genotype 2 plus 4; NI: not informed.
